# Optimization and Validation of Procyanidins Extraction and Phytochemical Profiling of Seven Herbal Matrices of Nutraceutical Interest

**DOI:** 10.3390/antiox13050586

**Published:** 2024-05-10

**Authors:** Niloufar Keivani, Vincenzo Piccolo, Adua Marzocchi, Maria Maisto, Gian Carlo Tenore, Vincenzo Summa

**Affiliations:** Department of Pharmacy, School of Medicine and Surgery, University of Napoli Federico II, Via Domenico Montesano 49, 80131 Napoli, Italy; niloufar.keivani@unina.it (N.K.); vincenzo.piccolo3@unina.it (V.P.); adua.marzocchi@unina.it (A.M.); maria.maisto@unina.it (M.M.); giancarlo.tenore@unina.it (G.C.T.)

**Keywords:** procyanidin, flavonoid, medicinal herbs, extraction, HPLC-HESI-MS/MS analysis, antioxidant

## Abstract

Several medicinal herbal plants are extensively used as sources of bioactive compounds with beneficial effects on human health. This study assessed the procyanidin and polyphenol profiles together with the antioxidant potential of seven herbal medical matrices. To achieve this aim, procyanidin extraction from grape pomace was optimized and validated by monitoring monomeric-trimeric procyanidins. The proposed quantification method was applied to the seven medical herbs, and it proved to be a very efficient protocol for procyanidin-rich extracts analysis. In addition, the *Paullinia cupana* Kunth. seed was identified as a very rich source of procyanidins (about 5 mg/g dry matrix of each dimeric and about 3 mg/g dry matrix trimeric) with high antioxidant properties. The polyphenolic profile was assessed by HPLC-HESI-MS/MS analysis. The in vitro antioxidant activity was evaluated by DPPH assay to explore the antioxidant properties of the extracts, which were substantially higher in *Peumus boldus* Molina leaves extracts (935.23 ± 169 μmol of Trolox equivalent/g of dry weight) concerning the other matrices. Moreover, a high Pearson coefficient value was observed between the total flavonoid content (TFC) and DPPH in comparison with the total polyphenol content (TPC) and DPPH, indicating flavonoids as the principal bioactive with antioxidant activity in the extracts.

## 1. Introduction

Medicinal herbal plants represent a rich source of bioactive compounds with beneficial effects on human health [1]. In this study, attention was focused on proanthocyanidins, also known as condensed tannins, which were found in many plant sources, including flowers, seeds, nuts, fruits, and barks. Condensed tannins are oligomers or polymers of flavan-3-ol units [2]. They are divided into two classes, procyanidins—which are derived from the condensation of catechin or epicatechin units—and prodelfinidins—which are formed by the condensation of gallocatechin or epigallocatechin units [3]. Flavan-3-ols are characterized by two aromatic rings (A and B) and one pyran ring (C), with a C6-C3-C6 configuration. Procyanidin monomer units can be linked by 2β→O-7 and 4β→8 bonds, known as “A”-type, or by a 4β→8 bond, known as “B”-type, while the linkage between C-4→C-6 is known as “C-type” linkage [4]. These compounds are characterized by several biological activities, including anti-inflammatory, anti-diabetic, and anti-cholesterol activities. Procyanidins are characterized by potent antioxidant properties with a scavenging activity against a wide range of free radical compounds, such as reactive oxygen species (e.g., ROS) and reactive nitrogen species (e.g., RNS). The antioxidant activity is strictly related to the presence of phenolic groups on the A and B rings, and the C2−C3 double bond in C-ring. This activity was confirmed by several studies in which the scavenging activity against the 2,2-diphenyl-1-picrylhydrazyl free radical (DPPH) has been evaluated [5,6]. The antioxidant activity determines a reduction in human oxidative stress that is related to the development of several diseases. This property is corroborated by the reduction in the biomarkers of lipid peroxidation, oxidative DNA damage, and protein oxidation, which was observed in several clinical trials [7,8,9,10,11,12,13].

In addition to antioxidant activities, procyanidins act as anti-diabetic, anti-inflammatory, and anti-cholesterol agents, which was proved by in vitro assays, as well as in clinical trials [14,15,16]. For example, the dimeric procyanidins of Annurca apple (*Malus Pumilar* Miller. cv Annurca) control the intestinal cholesterol micellar solubility and the fecal cholesterol excretion [17]. In addition, oligomeric and dimeric procyanidins bind plasmatic high-density lipoproteins (HDLs), showing a favorable impact on the reverse transport and the metabolism of cholesterol [18]. Several in vitro and in vivo studies have reported procyanidins activity in the regulation of insulin sensitivity and resistance. For example, procyanidins regulate glucose uptake in hepatocytes, myotubes, and adipocytes by regulating the phosphorylation of AMPK and by promoting GLUT4 translocation to the plasma membranes [19]. In addition, procyanidins directly regulate the activity of several enzymes involved in glucose homeostasis (e.g., glycogen synthase, glucose-6-phosphatase, and fructose-1,6-biphosphatase) [20].

Procyanidins also play a protective activity role against inflammatory diseases. Several studies showed the effect of procyanidins on inflammatory mediators, including IL-1β, the inflammasome NLRP3 (NLP pyrin domain containing 3), the factor TLR4 (Toll-like receptor 4), cyclooxygenase 2 (COX2), and inducible nitric oxide synthase (iNOS) [21]. Thereby, the anti-inflammatory activities of A- and B-type procyanidins have been surveyed by multiple in vitro and in vivo assays [22,23], they are considered as promising natural compounds, able to drastically reduce or resolve the inflammation status [24].

The growing interest in herbal matrices has grown significantly in recent years for the development of nutraceuticals, cosmetical products, and herbal remedies [3,25]. In order to develop a general method for procyanidins extraction and analysis, different botanical parts (e.g., flowers, leaves, seeds, roots) were selected. In addition, our selection included herbal plants of nutraceutical interest that were included in the European BELFRIT list, which includes plants safe for the use in food supplements [26]. Among these herbal species, our interest was focused on seven herbal matrices, including *Paullinia cupana* Kunth. seeds, *Crataegus monogyna* Jacq. flowers and leaves, *Peumus boldus* Molina, *Hamamelis virginiana* L. leaves, *Eleutherococcus senticosus* Maxim. roots, *Sambucus nigra* L. leaves, and *Sambucus nigra* L. flowers.

*Paullinia cupana* Kunth., locally called guaraná, has been proven to have many pharmacological properties, like energy boosting, chemoprophylactic, anti-genotoxic, anti-depressive, anxiolytic, and anti-amnesic [27]. Hawthorn (*Crataegus monogyna* Jacq.) fruit or leaves extracts, as well as *Peumus boldus* Molina leaves with a range of alkaloidal and non-alkaloidal phenolic constituents, due to their intense antioxidant and free radical scavenging properties have been the subjects of several studies [28,29]. *Hamamelis virginiana* bark extracts have been used for controlling atopic dermatitis symptoms, varicose veins, and other skin disorders. In this study, we focused on its leaves for their potential tannin-containing extracts [30]. *Eleutherococcus senticosus* Maxim., renowned for its adaptogenic effects in oriental traditional medicine, has previously proved to have anti-inflammatory activities, specifically an anti-hyaluronidase and anti-tyrosinase activity [31]. European elderberry (*Sambucus nigra* L.), a deciduous shrub owing to the known anthocyanin content of branches and berries, and flavonols and cinnamic acids esters in branches exhibited strong antioxidant activities, introducing it as a source of natural antioxidants [32].

The wide range of biological activities of procyanidins in the mitigation of inflammatory responses and oxidative stress involved in several diseases has triggered interest in the development of innovative nutraceuticals. To achieve this goal, it is a primary objective to develop and validate accurate analytical methods for the screening and analysis of components in the food matrices of interest in order to link their biological activity with the compositions. Therefore, in order to assess the parameters for extracting procyanidins, an extraction method was optimized using grape pomace (*Vitis vinifera* L., Aglianico cultivar). In addition, a HPLC-FLD method was optimized for procyanidins quantification in herbal matrices.

The optimized parameters were validated to confirm the reliability of the procedure and the validity of observed data. The validation methods criteria included the precision, accuracy, recovery, and matrix effect. In addition to the procyanidin profile, the polyphenolic composition and antioxidant activity of the extracts were also determined by HPLC-HESI-MS/MS analysis and DPPH assay, respectively. To the best of our knowledge, up to today, between the herbal matrices selected, the procyanidin profile of the *Eleutherococcus Senticosus* Maxim. root has not been reported yet. In addition, this study represents the first investigation of the radical scavenging activity by DPPH assay of *Hamamelis virginiana* L. leaves and *Eleutherococcus Senticosus* Maxim. root extracts. The oligomeric procyanidin profile, the total polyphenol content (TPC), the total flavonoid content (TFC), and the DPPH inhibitory activity of the herbal extracts were exhaustively investigated. To assess the relationship between the antioxidant activity of herbal extracts and polyphenolic composition, a Pearson correlation test was performed to correlate the antioxidant activity with TPC, TFC, and each procyanidin compound content.

## 2. Materials and Methods

### 2.1. Reagents

(+)-Catechin (CAS: 154-23-4), Procyanidin B1 (CAS: 20315-25-7), Procyanidin B2 (CAS: 29106-49-8), Procyanidin A2 (CAS: 41743-41-3), and Procyanidin C1 (CAS: 37064-30-5), with purity ≥ 98%, were purchased from PhytoLab GmbH and Co. KG (Vestenbergsgreuth, Germany). (−)-Epicatechin (≥98%) (CAS: 17334-50-8), Gallic acid (≥98%) (CAS: 149-91-7), Folin–Ciocalteu reagent (≥99.9%), 2,2-diphenyl-1-picrylhydrazyl (DPPH) (≥99.9%), 6-hydroxy-2,5,7,8-tetramethylchroman-2-carboxylic acid (Trolox) (≥99.9%), Sodium carbonate (Na_2_CO_3_), Sodium nitrite (NaNO_2_), Aluminum chloride hexahydrate ((AlCl_3_)_6_H_2_O), Sodium hydroxide (NaOH), HPLC-grade methanol (≥99.9%), and acetonitrile (≥99.9%) were acquired from Sigma-Aldrich (Milan, Italy). Formic acid (98–100%) (CAS: 64-18-6) were supplied from Romil Ltd. (Cambridge, UK). Water was obtained by a Milli-Q water purification system (Millipore, Bedford, MA, USA).

### 2.2. Plant Material

Seven medicinal herbal plant parts, including *Paullinia cupana* Kunth. seeds (cultivated in Brazil, harvested all year around—except the sudden frost period), *Crataegus monogyna* Jacq. flowers and leaves cut (Europe, harvested during the spring), *Peumus boldus* Molina leaves (Chile, harvested during the spring), *Hamamelis virginiana* L. leaves (cultivated in France, harvested in September), *Eleutherococcus senticosus* Maxim. roots (cultivated in China, harvested in May and October), *Sambucus nigra* L. leaves (Hungary, harvested during the summer), and *Sambucus nigra* L. flowers (East Europe, harvested during the summer). All the herbal matrices were purchased as dried products from Farmalabor SRL (Canosa di Puglia, Italy). Grape pomace (*Vitis vinifera* L., Aglianico cultivar) was collected during the harvest period in autumn, from Avellino, Campania, Italy. After harvesting, grape pomace was freeze-dried. Before utilization, all the plant samples were ground with an analytical IKA A 11 basic mill (IKA Werke GmbH & Co. KG, Staufen, Germany) while care was taken to avoid overheating.

### 2.3. Extraction Protocol

#### 2.3.1. Experimental Design

The identification of independent variables that would significantly affect the yielding results is of main importance in the optimization of extraction methods. It could be achieved through an adaptive one-factor-at-a-time (OFAT) strategy [33]. This approach was preferred over other experimental design strategies because it allows us to explore the effects of individual factors on the overall response without adding the complexity of multiple variables at the same time [34]. In this study, this approach was conducted to evaluate the impacts of various parameters, including solvent composition (0–100% of methanol), the solid-to-liquid ratio (0–150%), solvent acid content (0–2% of formic acid), and the sonication and agitation time (0–20 min) on procyanidins extraction yields from grape pomace matrix. Each parameter was varied while the others were kept constant. The generated data would be promising prospective results to optimize other extraction techniques. Eventually, the significant variable values were taken into account to define an optimized extraction protocol, which was applied for the extraction and the evaluation of procyanidins content from other seven herbal plant matrices.

#### 2.3.2. Procyanidins Extraction

Procyanidins extraction was performed using the protocol previously described with slight modifications obtained by the results of the OFAT approach optimization [35]. The extraction procedure was performed as follows: 50 mg of lyophilized matrix was homogenized with 1 mL of 60% aqueous methanol (*v*/*v*) containing 1% of formic acid, sonicated for 10 min (continuous operative mode, 150 W Power, 40 kHz Frequency; Branson Fisher Scientific 150E Sonic Dismembrator, Branson Ultrasonics Corporation, Danbury, Connecticut, United States), and kept under shaking (600 rpm, 25 °C) (Sko-DXL, Argolab, Carpy, Italy) for 15 min. After centrifugation at 9000 rpm for 10 min, the supernatants were collected in another plastic tube. The pellets were re-extracted with 1 mL of the extraction mixture as previously reported, and the supernatants were combined. The organic solvent was evaporated, and the residue was freeze-dried and stored at −20 °C until analysis. All extractions were performed in triplicate. The extracts were dissolved in methanol at a concentration of 10 mg/mL for the qualitative and quantitative analyses and the antioxidant assays.

### 2.4. Qualitative and Quantitative Analyses

#### 2.4.1. Procyanidins Qualitative Characterization

A LTQ XL mass spectrometer (Thermo Fisher Scientific, San Jose, CA, USA), coupled with HPLC DIONEX UltiMate 3000 (Thermo Fisher Scientific, San Jose, CA, USA) equipment, coupled with an autosampler, a binary solvent pump, and a diode-array detector (DAD), was used for the qualitative analysis [36]. A Kinetex^®^ C18 column (75 mm × 2.1 mm, 2.6 µm; Phenomenex, Torrance, CA, USA) was selected for the separation. The mobile phases included water at 0.1% formic acid (A) and acetonitrile at 0.1% formic acid (B). Elution was performed as follows: 0–3 min maintaining at 5% solvent B, increments from 5% (B) to 95% (B) in 22 min, followed by 3 min of holding; in the left 3 min, the column was equilibrated to the initial conditions. The injection volume was 5 µL, the column temperature was set at 35 ℃, and the flow rate was 0.35 mL/min. For the mass parameters, the source was a heated electrospray interface (HESI), operated in negative ionization with full scanning (FS) and data-dependent acquisition (DDA). Collision-induced fragmentation created by argon, with a collision energy of 35.0 eV. The source operated in both positive and negative ionization modes for the analysis of the procyanidins-containing extracts. The positive ionization mode was applied by an ion source at the following conditions: auxiliary gas flow rate: 10; sheath gas flow rate: 30; source heated temperature: 150 °C; capillary temperature: 320 °C; source current: 100 µA; source voltage: 3.5 kV; tube lens: 80 V; and capillary voltage: 32 V. The ion source was set for negative ionization mode using the following parameters: auxiliary gas flow rate: 10; sheath gas flow rate: 30; source heated temperature: 150 °C; capillary temperature: 320 °C; source current: 100 µA; source voltage: 3.5 kV; tube lens: 90 V; and capillary voltage: 31 V. The qualitative analysis was performed on the optimized hydromethanolic extract prepared at a concentration of 0.025 mg/mL in a 60% aqueous methanol (*v*/*v*) solution containing 1% formic acid. The samples were filtered with a 0.22 μm nylon filter and stored at −20 °C until analysis.

#### 2.4.2. Procyanidins Quantitative Characterization

A HPLC Jasco Extrema LC-4000 system (Jasco Inc., Easton, MD, USA), coupled with an autosampler, a binary solvent pump, a diode-array detector (DAD), and a fluorometric detector (FLD) was used for the analysis. The chromatographic analysis was performed as previously described [37]. The separation was achieved using a Kinetex^®^ C18 column (250 mm × 4.6 mm, 5 µm; Phenomenex, Torrance, CA, USA). The mobile phase consisted of two phases including water at 2% of formic acid (A) and a mixture of water–acetonitrile (49.75:49.75 *v*/*v*) with 0.5% of formic acid (B). The injection volume was 20 µL, the flow rate was kept at 1 mL/min, and the column temperature was at 30 °C. The elution gradient was: 0–2 min 10% (B), followed by a linear increase in solvent B to 55% up to the 50th min and then increasing up to 95% up to the 60th minute. The column was washed at 95% of solvent B for five minutes and reconditioned to the initial condition for five minutes. Procyanidins were monitored by the fluorescence detector (excitation wavelength: 272 nm; emission wavelength: 312 nm) and peak identifications were performed by a comparison of the retention times with analytical standards through co-injection with authentic standards. The procyanidin group of compounds was quantified according to the calibration curves (*R*^2^ ≥ 0.99) generated with six different concentrations (concentration range: 0.5–100 ppm) of related standards solubilized in methanol and triplicate injections at each point.

### 2.5. Validation of the Extraction Protocol

The extraction method validation was performed by the estimation of method recovery and the matrix effect, which can influence the sensitivity and reproducibility of the established chromatographic method, along with the ionization efficiency and retention time [38]. Acceptable recoveries range from 70 to 120%. The extraction efficiency was evaluated by the term of recovery percentage (R%). In this regard, the spiking procedure was utilized; the grape pomace samples were extracted by the addition of three levels of concentration of the procyanidins into solvents (pre-spike samples). Also, post-spike samples were prepared after the regular extraction process [39]. Considering the lack of procyanidin A2 in the grape pomace extracts, it was evaluated in *Paullinia Cupana* Kunth. seeds extracts. Recoveries were calculated as follows (Equation (3)):

(1) $$\mathrm{Recovery}=(\frac{\mathrm{area}\;\mathrm{analyte}\;\mathrm{in}\;\mathrm{pre-spike}\;\mathrm{sample}\;}{\mathrm{area}\;\mathrm{analyte}\;\mathrm{in}\;\mathrm{post-spike}\;\mathrm{samples}})\;\times \;100$$

Ultimately, with the intention of evaluating the effects of other present compounds in the sample matrices on the method efficiency, the matrix effect percentage (ME%) was estimated following the equation below [40]:

(2) $$\mathrm{ME}\;\%=(\frac{\mathrm{area}\;\mathrm{of}\;\mathrm{post-extraction}\;\mathrm{spike}\;}{\mathrm{area}\;\mathrm{of}\;\mathrm{standards}}-1)\;\times \;100$$

### 2.6. Validation of the HPLC-FLD Analysis

The validation of the procyanidins HPLC analytical method was attained according to the guidelines proposed by The International Council for Harmonization of Technical Requirements for Pharmaceuticals for Human Use (ICH) (Harmonized Tripartite guideline (Q2 [R1], 1995)) and the Food and Drug Administration (FDA) (November 2005) to confirm the reliability of the developed method (ICH, 2005). The analytical method undergoes several assessments to indicate the level of its effectuality on routine laboratory trials. In this regard, the parameters of linearity, the limit of detection (LOD), the limit of quantification (LOQ), repeatability—which is also termed intra-day precision assay—intermediate precision (inter-day precision assay), and accuracy were measured for each procyanidin compound as the standard methanolic solution [41].The limit of detection (LOD) and the limit of quantification (LOQ) of six compounds(e.g., catechin, epicatechin, procyanidins B1, B2, A2, and C1) were determined as the signal-to-noise ratio (S/N) of 3 and 10, respectively [42]. Accuracy and precision were determined through intra-day and inter-day assays; the intra-day process was carried out by three sets of standard solutions (n = 3) in different concentrations analyzed on the same day, while the inter-day analysis was established on three consecutive days. The coefficient of variation (% CV) which is estimated through the division of standard deviation with mean value was used to indicate the precision (Equation (1)) [43].

Precision = 100 [%] − CV(3)

Accuracy (trueness), representative of the proximity of compliance between experimental and expected values, is expressed as bias (%) which is calculated through the variation of the “measured value” (x) and the “true value” (µ_T_), (Equation (2)); the range of valid bias limits varies depending on the type and objectives of the analysis [44].

Bias = x − µ_T_ (4)

### 2.7. Total Polyphenol Content

The total phenol content was determined using the Folin–Ciocalteu method through adding 0.125 mL of Folin–Ciocalteu reagent, 0.5 mL distilled water to 0.125 mL of the diluted extract, prepared by using the collected supernatant that was kept at 4 ℃, which was diluted by the fitting dilution ratios with methanol, followed by the addition of 1.25 mL of 7.5% (*w*/*v*) sodium carbonate (Na_2_CO_3_) aqueous solution after 6 min. Following the 90 min resting at room temperature in the dark, the absorbance was measured at 765 nm using a UV-visible spectrophotometer (Jasco Inc., Easton, MD, USA). The estimation of total phenolic compounds in the extracts was carried out in quadruplicates. Blanks were prepared by the substitution of the samples with distilled water. Gallic acid was used to prepare a calibration standard curve (*y* = 3.7993*x* − 0.0077, *R*^2^ = 0.99) using 10 different concentrations in methanol ranging from 0.01 to 600 ppm, and dilution factors of 2 and 10 with three replicates at each concentration. The results were expressed as milligrams of gallic acid equivalents per gram of dry weight of the herbal matrix (mg GAE/g DW) [45].

### 2.8. Total Flavonoid Content

To assess the total flavonoid content, a modified colorimetric method was applied [46]. In brief, 0.25 mL of the hydromethanolic extract diluted with 1.25 mL of distilled water was added to 0.075 mL of 5% *w*/*w* sodium nitrite (NaNO_2_) aqueous solution, following 5 min rest in the dark. Then, 0.15 mL of 10% *w*/*w* aluminum chloride hexahydrate ((AlCl_3_)_6_H_2_O) aqueous solution was added. Lastly, 0.5 mL of 1 mol/L (4% *w*/*v*) sodium hydroxide (NaOH) aqueous solution was added to the mixture, followed by 6 min of staying in the dark. The final total volume was brought to 2.5 mL using distilled water. The solution was blended well prior to immediate absorbance measurement against the blank at 510 nm using a spectrophotometer (Jasco Inc., Easton, MD, USA). The results were expressed as the mg of catechin equivalents. Catechin was used to prepare a calibration standard curve (*y* = 2.9413*x* − 0.0379, *R*^2^ = 0.99) using 7 concentrations in methanol ranging from 0.1 to 250 ppm, and dilution factors of 2 and 5 with three replicates at each concentration. The results were expressed as milligrams of catechin per gram of dry weight of the herbal matrix (mg CAT/g DW).

### 2.9. Antioxidant Activity

To assess the antioxidant activity of the extract samples, the DPPH radical scavenging activity estimation assay was performed with slight modifications [47]. In total, 0.2 mL of a solution of each procyanidin-containing extract sample was mixed with 1.0 mL of a methanolic DPPH working solution 0.05 mM, prepared by the dilution of DPPH stock solution (1 mM) in methanol, and the mixture was allowed to react for 10 min at an ambient temperature in the darkness. The absorbances at 517 nm (A_2_) were measured using distilled water as the blank and methanol for the autozero. The DPPH radical scavenging activity was calculated as follows:

DPPH radical scavenging (%) = [(A_1_ − A_2_)/A_3_] × 100 (5)

where A_1_ is the absorbance of the sample at t = 0, A_2_ is the absorbance of the sample after the reaction time and A_3_ is the absorbance of the control. The obtained results are expressed in µmol of Trolox (6-hydroxy-2,5,7,8-tetramethylchroman-2-carboxylic acid) equivalent (TE). A calibration curve was generated by plotting the concentrations of Trolox standard against absorption at 517 nm, and the Trolox equivalent concentrations in samples were calculated from the derived linear equation (*y* = 0.3646*x* − 2.6699, *R*^2^ = 0.99) resulting from eight concentrations, in the range of 5–250 µM, and three replicates at each concentration. The results were expressed as μmol of Trolox equivalents per gram of dry weight of the herbal matrix (μmol Trolox equivalent/g DW).

### 2.10. Statistical Analysis

All experiments and tests were conducted in triplicate/quadruplicate. The data are expressed as the mean ± standard deviation (SD). The statistically significant differences among the samples were analyzed by one-way analysis of variance (ANOVA) using SPSS 28.0 software (SPSS Inc., Chicago, IL, USA). Differences with *p*-values < 0.05 were considered statistically significant. The Pearson correlation plot was obtained using total polyphenols and total flavonoids and procyanidins quantitative data and antioxidant assay results. PCA, Pearson correlation coefficients, and Pearson correlation plot was generated using OriginPro 2021b (OriginLab Corporation, Northampton, MA, USA). Variables were normalized using an autoscaling of data.

## 3. Results and Discussion

### 3.1. Extraction Optimization

#### 3.1.1. Evaluation of the Solvent Effects for Procyanidins Extraction

**Figure 1:**
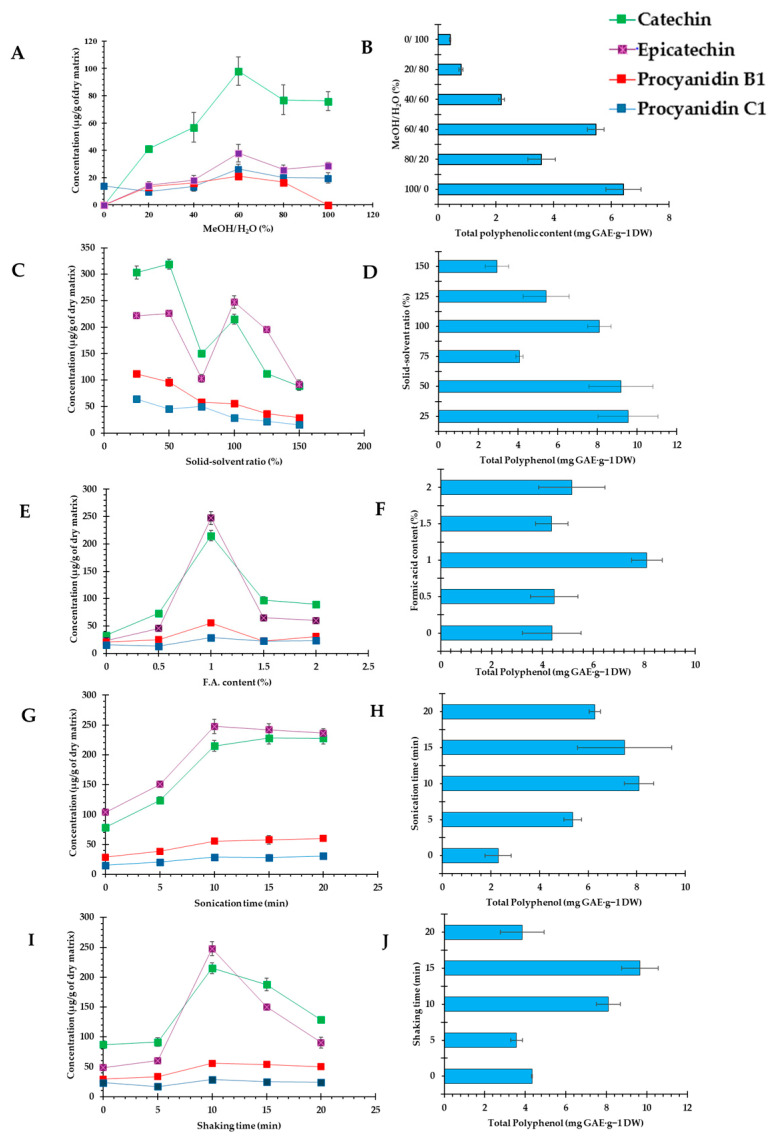
Evaluation of the different parameters on procyanidin distribution and total polyphenols content in grape pomace extracts. Graphical interpretation of the results calculated as mean ± SD. (**A**,**B**): evaluation of the solvent composition (MeOH/H_2_O %); (**C**,**D**): evaluation of the solid–solvent ratio (%); (**E**,**F**): evaluation of the formic acid content (%); (**G**,**H**): evaluation of the sonication time (min); (**I**,**J**): evaluation of the shaking time (min).

Extraction represents the preliminary step for the quantification of bioactive compounds in a food matrix. In this study, the experimental screening design focused on the impact of five extraction factors, which include the polarity of the solvent, solid/solvent ratio, acid content, ultrasound treatment, and agitation lengths. To assess procyanidins extraction, grape pomace was selected as a well-known source of a vast type of procyanidins [48]. As the solvent composition is of importance in the extraction of procyanidins, which is a flavonoid subclass, different hydromethanolic combinations were applied. Due to the hydroxyl groups existing in the structures of flavanols, moderate polar extractants are necessary for their solubilization. In addition, the use of water would facilitate their desorption from the plant matrix. The polarity index of water and methanol is 10 and 5.1, respectively [49]. The optimal polyphenol extraction efficiency resulting from the combination of an organic solvent with water, by the reduced level of polarity, has been reported in several studies [50,51,52]. To obtain the most efficient solvent composition, an initial single factor experiment considering the solid-to-solvent ratio of 100:1 (*w*/*v*) using grape pomace, with 10 min sonication and agitation time was performed. The results (Figure 1A,B, Table S1) indicated that 60% methanol was less effective with respect to the polyphenol content extraction, but the combination containing 60% methanol had a significantly higher efficiency of monomeric and dimeric in contrast to the pure methanol and water. The dimeric procyanidin extraction decreased significantly by the reduction in the methanolic portion. The significant high values of extraction obtained by 60% methanol are in agreement with previous results obtained which are suggested to be a result of improved solvent stability [53]. Although water has the highest polarity, it could not be used alone because its higher viscosity would have a detrimental effect on the mass transfer and the solubility of bioactive compounds. Hence, it would be a better option to be used in combination with alcohol, possessing a higher dielectric constant, to act as the plant swelling agent that facilitates penetration of the solvent to the internal plant structure [54]. Subsequently, in this study, the solvent containing 60% methanol was used to observe the effect of the other extraction parameters.

#### 3.1.2. Evaluation of the Solid-to-Solvent Ratio Effects for Procyanidins Extraction

The effect of the solid-to-solvent ratio on the procyanidin profile and total polyphenol content of grape pomace was investigated (Figure 1C,D, Table S2). Apparently, the rise of the solid-to-solvent ratio has a reverse impact on the TPC and procyanidin content. The TPC significantly decreased by 69% with the increment of the ratio up to 150% (*p* < 0.05). This could be the consequence of a lack of sufficient solvent for the solubilization of the food matrix and a less bioactive release rate. The same phenomenon could be observed in the quantified monomeric procyanidins present in the samples as they had a significant 70% reduction rate by raising the mass content, observed between the samples prepared by the solid-to-solvent ratio of 25% and 150%. The same reduction trend was observed for the procyanidin B1 by increasing the solid solvent ratio up to 125%. Although the trimeric procyanidin content declined slightly, a significant change was observed by 100% of the solid–solvent ratio. A similar trend was also observed in another study [55], where it was suggested to be resulted from the saturated utilized solvent by the addition of a higher biomass content which led to the least amount of phenolic compounds (by a 30–80% reduction rate). In addition, as was also anticipated by mass transfer principles, the declined concentration gradient happens by a higher solid-to-solvent ratio, terminating the diffusion process in the cellular ambiance.

#### 3.1.3. Evaluation of the Acid Content Effects for Procyanidins Extraction

Several percentages of formic acid (from 0 to 2%) was evaluated, showing that up to 1% of the presence of acid significantly helped procyanidins extraction and increased the polyphenolic content (Figure 1E,F, Table S3). The extractions with a 60% hydromethanolic mixture containing 1% formic acid presented the highest TPC extraction yield among the other solvents (8.10 mg GAE/g DW, *p* < 0.05). Acidified solvents are known to be more functional mediums for releasing polyphenols, because of their higher stability and neutral structure at low pH [56]. Exceeding acid content had a significant increase (*p* < 0.05) in the yields of procyanidins, as was observed in the TPC values. Apparently, the behavior of procyanidins alters depending on the solvent type and level of its acid content. In this study, a higher content of formic acid would negatively affect polyphenols due to their instability in strongly acidic conditions (from 8.10 ± 0.60 mg GAE/g DW to 5.15 ± 1.30 mg GAE/g DW, *p* < 0.05). Similar trends were observed for the quantitative analysis of procyanidins, having a raise up to 1% following significant reduction by adding up the formic acid content. For example, catechin and epicatechin displayed a significant content increase (*p* < 0.05) from 32.84 ± 3.40 μg/g DW to 215.00 ± 9.28 μg/g DW and 23.03 ± 1.90 μg/g DW to 247.56 ± 11.82 μg/g DW, respectively. The addition of more formic acid in the solvent induced the reduction in monomeric and dimeric procyanidins significantly, while no significant alteration was observed in C1 procyanidin content of the extracts. The results agreed with the literature data about the effect of pH modification in polyphenols extraction. For example, it was previously reported that quercetin-based flavonols extracted from grape skin or strawberry by-products were not stable in acidic methanol (containing formic, acetic, citric, and maleic acids) and were extremely labile in 1% hydrochloric acid while the addition of hydrochloric acid (0.5–1%).

#### 3.1.4. Evaluation of the Ultrasound Effects for Procyanidins Extraction

Ultrasound-assisted extraction methods have been developed to reduce the cost, time, and energy consumption in addition to providing environmentally friendly processes by reducing the used solvents [57]. Sonication up to 10 min significantly increases the TPC (from 2.30 ± 0.54 mg GAE/g DW to 8.10 ± 0.60 mg GAE/g DW, *p* < 0.05) and procyanidins extraction efficiency (Figure 1G,H, Table S4). The extension of ultrasonication time intensely influenced the monomeric procyanidins extraction, which amplified the amount of catechin (250%) and epicatechin (167%) from 78.62 ± 2.31 μg/g DW and 103.84 ± 2.21 μg/g DW to 228.00 ± 9.71 μg/g DW and 241.99 ± 9.75 μg/g DW, respectively, up to 15 min. However, sonication up to 20 min does not significantly improve procyanidins extraction, demonstrating that the solvent system is dynamically equilibrated. Therefore, 10 min of ultrasound treatment time was determined as the optimum condition for procyanidins extraction, as there was no significant increment in the results afterwards. Similar results were reported for polyphenols extraction from *Allium senescens* L. seeds, determining a significantly increasing impact on the yield up to 15 min and then a static trend of results [58]. Similarly, in another study, it has been stated that the extension of ultrasonication timing enhanced the total flavonoid extraction yield up to a specific timing (45 min), and then the reversed impact of ultrasonication was observed [59]. In fact, the initial cavitation based on ultrasonication assists the cell wall rupture, and in parallel, the rate of flavonoid releasing rate. Eventually, the membrane concentration equilibrium, impurities contents, and even oxidation happen as a matter of prolonged timing.

#### 3.1.5. Evaluation of the Shaking Time Effects for Procyanidins Extraction

Shaking represents a key factor in extraction optimization as it plays a role in the mass transfer equilibrium of the analytes to the extraction solvent phase. The physicochemical properties of phenolic molecules have a corresponding relationship with extraction time. For example, the polymers with a stronger linkage to the cell wall require an extended extraction time. Moreover, the solvent needs to have sufficient time to interact with the solid material until it reaches the mass transfer equilibrium point [60]. As shown in Figure 1I,J and Table S5, there is a significant variation in the extraction values of TPC up to 15 min (9.65 ± 0.89 GAE/g DW). The extraction efficiency of monomeric procyanidin content is almost doubled up to 10 min of shaking and there was no significant difference in catechin extraction yields with 10 min and 15 min of agitation, which are reported to be 215.00 ± 9.28 µg/g DW and 187.71 ± 10.86 µg/g DW, respectively. Likewise, the same trend is observed for epicatechin, with values of 247.56 ± 11.82 µg/g DW and 150.29 ± 2.84 µg/g DW. Meanwhile, there were no significant alterations in dimeric and trimeric procyanidins in the range of 10–20 min. Overall, present assessments indicated that 15 min with a stirring rate of 600 rpm at 25 °C could be the optimal agitation time for the representative compounds.

### 3.2. Procyanidin Extraction from Medicinal Herbal Plant Matrices

#### 3.2.1. Procyanidin Qualitative Analysis

Based on the quantitative optimization, the 60% hydromethanolic mixture with 1% formic acid, a solid-to-solvent ratio of 25:1 (*w*/*v*), the ultrasound treatment for 10 min and the 15 min stirring time was determined as the best method for procyanidins extraction. Therefore, this method was applied for the extraction of seven different medicinal plants (*Paullinia cupana* Kunth. seeds, *Crataegus monogyna* Jacq. flowers and leaves, *Peumus boldus* Molina leaves, *Hamamelis virginiana* L. leaves, *Eleutherococcus senticosus* Maxim. root, *Sambucus nigra* L. leaves and flowers), currently being under assessment for various nutraceutical purposes. In addition, this study filled the gap of a lack of optimization of procyanidins extraction parameters from these matrices. Following the optimization of procyanidins extraction, the qualitative analysis of the herbal plant matrices extracts was achieved by HPLC-HESI-MS/MS displaying the chromatogram and spectrums in positive and negative modes. The total polyphenolic putatively qualitative profile is shown in Table 1, including the retention times (Rt), the parent ions (*m*/*z*), and the fragmentation patterns (base peak ions are shown in bold). Procyanidins belong to the group of flavan-3-ols, a subclass of flavonoids that are characterized by a 3-hydroxy-3-phenylchromen-4-one scaffold [37]. Flavonoid provided reasonably intense negative and positive ions, leading to their structural recognition information about their structures. The flavan-3-ols were detected both in negative and positive ion mode spectrums in herbal samples. The interflavan linkage is the distinctive factor in the classification of procyanidins into two groups (A-type or B-type). B-type procyanidins contain (epi)catechin units bonded by C4-C8 or C4-C6, the [M + H]^+^ *m*/*z* values are 2 Da higher than the related A-type procyanidin. Based on the literature data [61], three fragmentation routes are involved in procyanidins ionization in negative HPLC-HESI-MS/MS analysis: the Retro Diels–Alder (RDA) and heterocyclic ring fission (HRF) reactions, which would result in the detection of the ions [M-136-H]^−^ and [M-125-H]^−^, respectively, and the quinine methide (QM) reaction, which fragments the interflavan bond with the detection of the fragment ions [M-289-H]^−^ or [M-287-H]^−^. QM fragmentation was usually observed in this study. It is noteworthy to mention that, depending on the degree of polymerization (DP), the numbers of isomers with the identical molecular weight of each procyanidin may exist in the matrix. In our study, seven different types of oligomeric procyanidins, alongside monomeric procyanidins were putatively identified, including: dimeric B-type and A-type, trimeric B-type and A-type, tetrameric B-type and A-type, and a pentamer derivative form of procyanidin (monogallate form of cinnamtannin A3). Procyanidin C1 displayed a [M−H]^−^ at *m*/*z* 865, which was detected in *Crataegus monogyna* Jacq. flowers and leaves extract analysis. Alongside this, procyanidin B2 had a molecular ion peak [M−H]^−^ at *m*/*z* 577 and MS^2^ fragment ions with *m*/*z* 457, 413, and 293 [62]. The parent ion, with *m*/*z* 865 ([M−H]^−^), is representative of procyanidin C1, whose fragmentation includes the fragment ions with *m*/*z* 847, 739, 695, 577, 451, 287. The fragment ion with *m*/*z* 695 resulted due to the RDA, derived by the consequential neutral loss of water [M−H-152-H_2_O]^−^ and the ion *m*/*z* 289 derived by the fragmentation of two flavanols monomers with QM fragmentation [63]. Malvidin 3-glucoside flavene–epicatechin dimer, a peculiar A-type dimeric procyanidin, was identified in the samples of *Eleutherococcus senticosus* Maxim. root and displayed a *m*/*z* 781 and fragment ions with *m*/*z* 631, 630, 586, 472. A-type procyanidins (dimeric A-type procyanidin with *m*/*z* 575, trimeric A-type procyanidin with *m*/*z* 863, and tetrameric A-type procyanidin with *m*/*z* 1151) were observed in negative ion mode spectrum of *Paullinia cupana* Kunth. samples. For example, cinnamtannin B1 (A-type procyanidin trimer) displayed a precursor ion peak [M−H]^−^ at *m*/*z* 863 and product ions of 711, 693, 573, 451, and 411 [64]. The positive ion mode mass spectrum displayed a parent ion with *m*/*z* 867 in positive acquisition mode ([M + H]^+^) and with *m*/*z* 865 in negative acquisition mode ([M−H]^−^), confirming the detection of trimeric procyanidins B-type linkage in *Crataegus monogyna* Jacq., and *Peumus boldus* Molina extracts. A protonated procyanidin molecular ion [M + H]^+^ at *m*/*z* 1595 putatively indicates a pentamer monogallate yielded from *Sambucus nigra* L. flower extracts. Along with the procyanidins other polyphenolic compounds were also detected through the MS analysis, including gallotannins (Hydrolyzable tannins) and flavonoids. Gallotannins represent an ester form of gallic acid in combination with polyols or glycosides mostly containing β-D-glucopyranose. They were detected in *Hamamelis virginiana* L. leaves extracts, which have been proven to have high antioxidants [65]. The mass spectra allowed us to tentatively identify 8 various flavonols in herbal matrices: quercetin-*O*-hexuronide, quercetin-*O*-deoxyhexoside, quercetin-*O*-galactoside, quercetin-*O*-glucoside, quercetin-*O*-dihexoside, quercetin-*O*-rhamnosylglucoside, and quercetin-*O*-deoxyhexose–hexose. All these compounds displayed a diagnostic fragment ion with *m*/*z* 301, which represented the quercetin aglycon moiety. The flavonoid glycosides derived from the aglycone of kaempferol, such as kaempferol pentosyl-dirhamnoside and kaempferol-*O*-glucoside, were identified in *Peumus boldus* Molina leaves and *Crataegus monogyna* Jacq. leaves and flower samples. The fragmentation pattern of these compounds includes the fragment ions with *m*/*z* 285 (negative ion mode) and *m*/*z* 287 (positive ion mode).

#### 3.2.2. Procyanidin Quantitative Analysis

Bioactive compounds are generally found in plants at low concentrations. Therefore, their recovery at high yields with minimal changes in their structure would be dependent on the extraction procedure and would be influenced by a range of factors, like the polarity of the solvent, pH, the extraction time, and the plant material composition. Regarding this, for quantification of monomeric and oligomeric procyanidins in extracts prepared with mentioned herbal matrices, optimized extraction and validated HPLC methods have been used. The identification and quantification of compounds were conducted by retention times of the chromatographic peaks and comparison with analytical standards. The quantitative results are summarized in Table 2. Fluorescence absorption and emission spectra indicate optimal measurement at an excitation wavelength of 272 nm and an emission wavelength of 312 nm. Fluorescence detector (FLD) was preferred to other detection systems (e.g., diode-array detector DAD and UV) for the greater sensitivity and selectivity for procyanidins analysis. Moreover, UV detection is not specific for procyanidins in presence of other polyphenols [98]. Among the monomeric units, catechin was found to be more abundant in all matrices except for the extract of *Crataegus monogyna* Jacq., in which it was not detected. The highest content of catechin was recovered from *Paullinia cupana* Kunth. seeds extract (36,153 µg/g DW). Nonetheless, a great amount of epicatechin in the extract obtained from *Crataegus monogyna* Jacq. leaves and flowers was remarkable to notice (≈4607 µg/g DW). To our knowledge, up to today, A-type dimeric procyanidin was never quantified in *Crataegus monogyna* Jacq. leaves and flower extracts (351.5 µg/g DW). Among all the herbal matrices, B1 procyanidin was plentifully observed in *Peumus boldus* Molina leaves extracts (33,874 µg/g DW). In addition, to the best of our knowledge, the procyanidin B1 occurrence in *Hamamelis virginiana* L. leaves is also reported for the first time in this study (2340 µg/g DW). Predominantly, procyanidin A2 was rich in *Paullinia cupana* Kunth. seeds extract (5075 µg/g DW), which agreed with previously published results extracted with a 70% aqueous methanol as solvent (≈13 mg/g extract) [99]. Trimeric procyanidin was abundantly found in three matrices (*Crataegus monogyna* Jacq. extracts, *Peumus boldus* Molina leave, *Paullinia cupana* Kunth. seeds) while low values or a lack of it were observed in the other matrices.

### 3.3. HPLC-FLD Method Validation

The HPLC-FLD method for procyanidins quantitative analysis has been validated by the assessment of linearity, the limits of detections (LODs), the limits of quantification (LOQs), the precision, and the accuracy. The analysis included the quantified flavanols compounds of this study (e.g., catechin, epicatechin, and procyanidins B1, B2, and C1). Calibration curve parameters are summarized in Table 3. The calibration curves were calculated using the linear regression model. A favorable linear regression factor (*R*^2^ > 0.99) was calculated for all quantified compounds in the range of 0.5 to 100 µg·mL^−1^ of all the monomeric, dimeric, and trimeric procyanidins. The sensitivity of the HPLC-FLD method was confirmed by the low LODs and LOQs values, which ranged from 42.8 ± 6.47 ppb to 280.0 ± 5.26 ppb and from 128.40 ± 19.41 ppb to 840.00 ± 15.78 ppb, respectively.

The HPLC-FLD method has been validated through intraday and interday variability approaches for the assessment of precision (coefficient of variation %CV) and accuracy (% bias). The analysis was performed at three different concentration levels (low: 1 μg/mL; medium: 10 μg/mL; high: 100 μg/mL). The results are summarized in Table 4. Procyanidins intraday precision was assessed as ≤10%, while in interday analysis, the range was slightly higher (≤15%). In particular, the higher % CV intraday values were observed at low concentrations for dimeric and trimeric procyanidins (procyanidin A2% CV at 1 μg/mL: 10.3; procyanidin C1% CV at 1 μg/mL: 8.8). Similarly, the higher % CV interday values were observed at low concentrations for dimeric procyanidins (procyanidin B1% CV at 1 μg/mL: 13.9; procyanidin B2% CV: 12.8). Procyanidins accuracy (% bias) ranged from −4.00 to 0.07 and from −4.21 to 0.08 for intraday and interday precision, respectively. Based on the low interday and intraday % bias and % CV values at all standards concentration levels, the HPLC-FLD method can be considered accurate for procyanidins quantification. In addition, validation results agreed with FDA and modified Westgard regulations, which reported % CV values not exceeding 20% [100].

### 3.4. Extraction Method Validation

Recovery and matrix effect experiments were performed for the assessment of the optimized extraction procedure (Table 5). The analysis included the quantified flavanols compounds of this study (e.g., catechin, epicatechin, and procyanidins B1, B2, and C1) and was accomplished by the addition of three levels of standards (high, medium, low) to the grape marc samples. As procyanidin A2 is not reported in the grape marc sample, *Paullinia cupana* Kunth. seeds were used to assess the recovery rate of this compound. The average recovery for all procyanidin compounds was between 79.43% and 112.02% for the tested working concentrations of the analytes, in accordance with guidelines for a good recovery assay [101]. Recovery values close to 100% confirm the effectiveness of the protocol for procyanidins extraction and suggest a high stability of these compounds in the optimized extraction conditions. These data are in agreement with literature results about procyanidins stability with an ultrasound-assisted extraction with hydroalcoholic solvents [102]. In addition, the matrix effect has been evaluated to assess the correctness and the reliability of the quantitative analysis, determining the influence of other sample components of procyanidins response in quantitative analysis. Although a matrix effect was observed in the analysis, values lower than 20% may be considered low and reliable for procyanidins quantification [103], with a % matrix effect in a range between −19.71 to 19.93%.

### 3.5. Total Polyphenol and Total Flavonoid Contents

The total polyphenol content and the total flavonoid content were evaluated by the colorimetric assay TPC and TFC. The polyphenolic extract of all medicinal herbal plant parts was prepared under optimized validated conditions. The TPC and TFC results of all the matrices are reported in Table 6. Among the herbal matrices, *Paullinia cupana* Kunth. seed, *Peumus boldus* Molina leaves, *Sambucus nigra* L. flower showed the highest TPC and TFC. The high TFC of leaves and flowers parts in the herbal samples can be explained by the flavonoid protective effect against UV radiation light, resulting from the higher sun radiation exposure time [104]. *Paullinia cupana* Kunth. seed extract showed the highest TPC and TFC results of 372.67 ± 15.49 mg GAE/g DW and total flavonoid of 42.59 ± 2.20 mg CAT/g DW, respectively. The high TPC and TFC content in *Paullinia cupana* Kunth. seed can be explained by the high quantified procyanidin A2 content. The TPC result observed for *Sambucus nigra* L. flower extract (134.10 ± 6.36 mg GAE/g DW) is higher than the aqueous, ethanolic, and hydroethanolic extracts values reported in the literature [105,106,107]. In an extraction optimization study on *Crataegus monogyna* Jacq. leaves using a solution of 50% acetone as extraction solvent, a TPC value (78.9 ± 0.4 mg GAE/g DW) similar to our result (71.31 ± 2.12 mg GAE/g DW) was observed [108].

### 3.6. Antioxidant Activity

A commonly employed method to assess antioxidant activity is the spectrophotometric assay of DPPH (2,2-diphenyl-1-picrylhydrazyl) radical scavenging. This reagent is classified as a stable free radical compound for the electron delocalization occurring in the whole molecule, preventing dimerization. The electronic delocalization is responsible for the violet color and absorption band at 517 nm of DPPH solution. In exposure with antioxidant compounds able to donate hydrogen atoms, the radicals are reduced. Therefore, a decrement of the absorbance at 517 nm would be monitored. Flavanols are capable of hydrogen donation and the neutralization of DPPH radical by the phenolic hydroxyl group [109]. Based on the literature data, the scavenging behavior of procyanidins depends on different factors including the source, the degree of polymerization, and the structure of the molecule. It has been suggested that A-type oligomeric procyanidins have a higher antioxidant activity in trimeric form in contrast to dimeric and monomeric. Meanwhile, the antioxidant capacity decreases over a degree of polymerization over 9 flavan-3-ol units. In general, it has been concluded that oligomeric procyanidins could be more potent than monomeric forms as DPPH radical scavenging compounds [110,111]. Due to the high procyanidin contents in the herbal matrices, the DPPH assay was selected for the evaluation of the antioxidant activity. The results of all the matrices are reported in Table 7 and are expressed as the equivalent of µmol of Trolox/g of dried herbal matrices. There is a parallel positive relationship between TFC and the exhibited antioxidant capacity. The significant highest values belong to *Peumus boldus* Molina leaves, *Sambucus nigra* L. flower, and *Paullinia cupana* Kunth. seed, with 935.23 ± 169.85 μmol Trolox/g DW, 758.89 ± 100.20 μmol Trolox/g DW, and 693.63 ± 48.04 μmol Trolox/g DW, respectively. Our results agreed with the literature data about the radical scavenging activity. The DPPH scavenging activities of *Sambucus nigra* L. flowers (in-house result: 758.89 ± 100.20 μmol Trolox/g DW—literature result: 570–920 μmol Trolox/g DW) [112], *Crataegus monogyna* Jacq. flowers and leaves (in-house result: 62.96 ± 5.27 μmol Trolox/g DW—literature result: 29–57 μmol Trolox/g DW) [113], and grape pomace (in-house result: 196.91 ± 0.86 μmol Trolox/g DW—literature result: 120–230 μmol Trolox/g DW) [114] were comparable to previously published data. Instead, the values of anti-radical activity of *Sambucus nigra* L. leaves (in-house result: 374.40 ± 47.20 μmol Trolox/g DW—literature result: 104.35 ± 0.22 μmol Trolox/g DW) [115], *Peumus boldus* Molina leaves (in-house result: 935.23 ± 169.85 μmol Trolox/g DW—literature result: 524.17 μmol Trolox/g DW) [28], and *Paullinia cupana* Kunth. seed (in-house result: 693.63 ± 48.04 μmol Trolox/g DW—literature result: 67.91 ± 4.00 μmol Trolox/g DW) [116] were significantly higher in our study compared to the literature data. The comparison with literature data is summarized in Table 7. These different values are probably due to the different extraction solvent (e.g., water and methanol without acidification) reported in the literature for polyphenols extraction compared to the in-house extraction solvent (e.g., aqueous solution at 60% methanol containing 1% of formic acid). In addition, to the best of our knowledge, the radical scavenging activity by DPPH assay of *Hamamelis virginiana* L. leaves and *Eleutherococcus senticosus* Maxim. root extracts have not been reported yet.

### 3.7. Pearson Correlation Analysis

**Figure 2:**
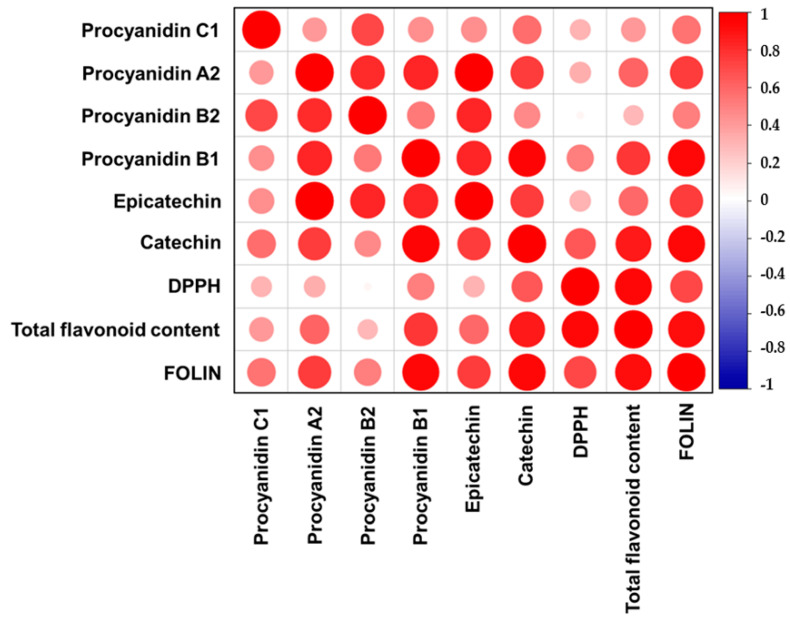
Pearson correlation plot of quantified procyanidins, TFC, TPC, and antioxidant assay values. The gradient in color and size of circle could be seen between the correlation values, with large red dots exhibiting the maximum positive correlation (+1); the smaller circles correspond to correlation coefficient close to 0 (indicating little to no correlation).

To assess the bioactivity of herbal extracts, a Pearson correlation test was performed to correlate the antioxidant properties with the content of polyphenolic compounds and the total flavonoids content (Figure 2, Table S6). The Pearson coefficient (*R*^2^) and significance (*p*-value) are greater between TFC and DPPH in comparison with the TPC (indicated as FOLIN) and DPPH values. This result suggests that the greater antioxidant activity is related to the flavonoids rather than to other FOLIN-responsive polyphenols (e.g., phenolic acid, hydroxycinnamic acid). Furthermore, the TFC strongly correlates with the catechin, epicatechin, and procyanidins A2 and B1 contents, confirming the effectiveness of the assay for estimating the content of total procyanidins. Furthermore, there are also indications of the antioxidant effectiveness of procyanidins. For example, there is a positive significant correlation between DPPH and catechin and procyanidin B1 content. However, the statistical correlation of the test compounds with DPPH does not give a strong Pearson coefficient, suggesting that procyanidins are only partially responsible for the scavenger activity. A non-significant statistical correlation (*p*-value > 0.05) was assessed by procyanidin B2 quantified in the samples with antioxidant assay value (DPPH/(µg Procyanidin B2/g DW), *R*^2^ = 0.04, *p*-value = 0.92). In contrast, procyanidin B1 was characterized by strong and significant positive correlation (DPPH/(µg Procyanidin B1/g DW), *R*^2^ = 0.51, *p*-value = 0.02). In addition, the Pearson correlation test proved a positive and significant correlation between the total flavonoid content and the DPPH, confirming procyanidins antioxidant activity (TFC/DPPH, *R*^2^ = 0.93, *p*-value = 0.001).

## 4. Conclusions

The single-factor approach was successfully used to reach the effective conditions of procyanidin extraction from seven herbal matrices along with the grape pomace. All the considered factors exhibited a significant effect on the TPC. The optimal conditions were a solid-to-solvent ratio percentage of 25%, a formic acid content of 1%, an ultrasound and agitation time of 10 min, and a methanol concentration of 60%. In comparison to the results obtained during OFAT optimization, the TPC, monomeric, dimeric, and trimeric procyanidin contents are significantly higher in the samples prepared by the optimized extraction method. In addition, TFC was assessed in the extracts obtained by this extraction protocol. A reliable method was performed for procyanidins quantification in grape pomace using the HPLC-FLD system. The extraction and analysis methods were assessed by the analysis of spiked samples through the method validation, and the achieved results regarding accuracy and precision were favorably accepted. Hence, the method described was sufficiently precise and accurate, and can be applied for the quantification of procyanidins in other herbal plant parts. Although the A-type procyanidin was absent in the reference matrix (grape pomace), it was distinctly quantified in *Paullinia cupana* Kunth. seeds and *Crataegus monogyna* Jacq. flowers and leaves. The monomeric (epicatechin), dimeric (procyanidin B2), and trimeric content in the latest was significantly at high levels (approximately about 5, 4, and 4 mg/g of dried matrix), respectively. The validated method was demonstrated to be appropriate for the routine scrutinization of oligomeric procyanidin. In addition, this study allowed for the full characterization of the procyanidin profile in the seven herbal matrices, showing *Paullinia cupana* Kunth. seeds and *Peumus boldus* Molina leaves as those with the highest content of procyanidins and antioxidant activity, respectively. The contribution of procyanidins to the antioxidant capacity of the extracts was evaluated, assessing a high positive correlation. Therefore, this study provides the basis for further investigation to assess the potential application of these herbal matrices for the development of novel nutraceutical formulations.

## Figures and Tables

**Table 1:** Qualitative analysis of the herbal medicinal matrices by HPLC-HESI-MS/MS in positive and negative acquisition mode.

Source Matrix	Rt (min)	[M−H]^−^ (*m*/*z*)	[M + H]^+^ (*m*/*z*)	Fragmentation Pattern	Putatively Compound	Reference
*Hamamelis virginiana* L. leaves	6.50	631.21	-	585.02, 479.03, 317.10	Myricetin-hexosyl-gallate	[66]
	6.67	-	611.16	449.15, 303.08	Cyanidin 3,5-diglucoside	[67]
	6.80	479.15	-	461.22, 317.12, 316.05	Myricetin-*O*-glucoside	[68]
	7.29	477.22	-	463.21, 300.97, 183.17	Quercetin-*O*-hexuronide	[69]
	7.46	939.13	-	787.17, 769.07, 617.25	Pentagalloyl glucopyranose	[70]
	7.67	447.23	-	327.13, 301.17, 285.14, 284.20	Quercetin-*O*-deoxy-hexoside	[71]
	7.76	1091.00	-	939.18, 769.25, 617.33	Hexagalloylglucose	[72]
	8.03	1243.18	-	1091.20, 939.24, 920.28, 769.26	Heptagalloylglucopyranose	[72]
	8.72	-	595.15	329.16, 309.10, 287.08	Cyanidin-3-rutinoside	[73]
*Peumus boldus* Molina leaves	6.17	-	867.05	715.36, 579.30, 577.13, 425.06, 287.15	Procyanidin C1 B-type linkage	[74]
	6.70	609.35	-	463.13, 447.12, 315.06, 301.03	Luteolin-glucopyranosil-glucopyranoside	[75]
	6.72	-	611.16	449.15, 303.08	Cyanidin 3,5-diglucoside	[67]
	7.06	623.12	-	595.24, 477.13, 461.18, 315.12	Isorhamnetin-*O*-rutinoside	[76]
	7.06	593.44	-	447.21, 431.30, 285.05	Isorhamnetin-*O*-deoxyhexosyl-pentoside	[75]
	7.41	709.29	-	563.19, 541.06, 431.19, 285.17	Kaempferol-*O*-pentosyl-*O*-dirhamnoside	[75]
	7.49	607.33	-	461.14, 315.13, 212.21	Isorhamnetin-*O*-dirhamnoside	[75]
	7.71	447.15	-	301.07, 299.99, 285.12	Luteolin-*O*-hexoside	[77]
*Crataegus monogyna* Jacq. flowers and leaves	6.21	-	867.22	715.11, 697.25, 579.22	Procyanidin C1 B-type linkage	[74]
	6.36	865.16	-	847.31, 739.19, 695.16, 577.21, 451.19, 287.17	Procyanidin C1 B-type linkage	[78]
	6.60	-	595.34	449.15, 431.16	(*Epi*)gallocatechin gallate B-type linkage	[79]
	6.68	-	449.39	431.08, 383.23, 329.20, 287.03	Kaempferol-*O*-glucoside	[80]
	6.72	593.21	-	473.15, 413.13, 293.13	Isorientin-*O*-rhamnoside	[81]
	7.04	-	579.31	433.24, 415.28	Procyanidin B2	[61]
	7.05	1155.12	-	1136.12, 1028.33, 984.19, 866.26,577.28, 407.18	Procyanidin tetramer B-type linkage	[61]
	7.05	577.28	-	457.23, 413.07, 292.98	Procyanidin B2	[62]
	7.26	463.12	-	301.02, 300.01	Quercetin-*O*-galactoside	[82]
	8.31	-	577.29	559.32, 425.20, 245.16	Procyanidin A2	[74]
	9.29	582.36	-	462.28, 436.36, 342.23	Tri-*p*-Coumaroyl spermidine	[83]
*Eleutherococcus senticosus* Maxim. root	7.30	463.40	-	331.18, 301.04, 161.010	Quercetin-*O*-glucoside	[84]
	9.46	809.43	-	791.22, 743.26, 647.42, 629.35, 471.23	malvidin 3-glucoside-ethyl-(epi)catechin	[85]
	9.46	663.42	-	645.52, 587.51, 487.27	Monoglucuronide methyl (-)-Epigallocatechingallate	[86]
	10.79	781.61	-	631.53, 630.43, 586.42, 472.44	Malvidin 3-glucoside flavene–epicatechin dimer A type	[87]
*Paullinia cupana* Kunth. seed	4.48	289.03	-	245.17, 205.12, 178.99	(*Epi*)catechin	[88]
	6.14	-	865.19	713.16, 695.20, 533.19	Procyanidin C1 A-type linkage	[89]
	6.36	863.29	-	711.11, 693.29, 573.18, 451.18, 411.15	Procyanidin trimer A-type linkage	[90]
	7.38	1153.27	-	1134.19, 1026.35, 864.15, 575.17, 423.19	Procyanidin tetramer B-type linkage	[91]
	7.38	575.08	-	539.12, 449.13, 423.20, 289.13	Procyanidin A2	[92]
	7.34	1151.37	-	1133.06, 1025.03, 999.10, 981.31, 863.21, 575.23	Procyanidin tetramer A-type linkage	[91]
*Sambucus nigra* L. flowers	6.74	625.25	-	505.12, 445.08, 300.08	Quercetin-*O*-dihexoside	[93]
	7.13	609.17	-	343.11, 301.03, 284.99	Quercetin-*O*-rhamnosylglucoside	[94]
	7.18	-	611.16	465.10, 303.02	Quercetin-*O*-deoxyhexose–hexose	[95]
	7.6	623.4	-	315.06, 300.10	Dihydroxy-dimethoxychalcone-*C*-diglycoside	[93]
	9.76	-	1595.76	1577.20, 1472.10, 1443.82, 1370.00, 613.47	Procyanidin pentamer monogallate A-type linkage	[96]
*Sambucus nigra* L. leaves	7.16	609.2	-	301.07, 300.03, 254.93	Quercetin-*O*-rhamnosyl-glucoside	[94]
	7.17	-	611.23	465.10, 303.05	Dimeric epigallocatechin (b type) or prodelphinidin dimer	[74]
	9.75	1593.67	-	1558.64, 1209.36, 821.60, 594.50	Procyanidin gallate pentamer A-type linkage	[97]

**Table 2:** Procyanidins content in medicinal herbal extracts.

Sample	Catechin	Epicatechin	Procyanidin B1	Procyanidin B2	Procyanidin A2	Procyanidin C1
	µg/g Dry Matrix					
*Sambucus nigra* L. flower	171.19 ± 4.11 ^a^	ND	166.78 ± 4.92 ^a^	64.53 ± 4.52 ^a^	ND	71.05 ± 1.96 ^a^
*Sambucus nigra* L. *leaves*	19.84 ± 1.50 ^a^	92.36 ± 8.90 ^a^	ND	228.55 ± 7.99 ^b^	ND	59.75 ± 3.77 ^a^
*Crataegus monogyna* Jacq. flowers and leaves	ND	4606.86 ± 271.42 ^b^	ND	4148.05 ± 235.18 ^c^	351.55 ± 35.59 ^a^	4040.59 ± 255.61 ^b^
*Peumus boldus* Molina leaves	27,694.7 ± 53.7 ^b^	1004.65 ± 62.60 ^c^	33,873.9 ± 67.84 ^b^	ND	ND	3169.23 ± 188.48 ^c^
*Eleutherococcus senticosus* Maxim. root	340.21 ± 23.96 ^c^	ND	ND	272.42 ± 27.92 ^d^	ND	ND
*Paullinia cupana* Kunth. seed	36,153.08 ± 2681.30 ^d^	36,267.49 ± 2596.96 ^d^	6593.71 ± 412.79 ^c^	5391.78 ± 350.42 ^c^	5075.61 ± 325.70 ^b^	2829.14 ± 126.94 ^c^
*Hamamelis virginiana* L. leaves	4547.11 ± 397.98 ^c^	ND	2339.66 ± 175.66 ^b^	ND	ND	ND
Grape pomace	345.83 ± 6.45 ^a^	238.26 ± 0.94 ^a^	169.72 ± 6.27 ^a^	144.90 ± 24.43 ^b^	ND	70.21 ± 0.95 ^a^

Results are expressed as mean ± SD, and *p* < 0.05 was considered statistically significant. Statistical significance was calculated by one-way ANOVA, followed by Tukey’s post hoc test. Different letters reveal significant differences. “ND” means “not detected”.

**Table 3:** Calibration curve parameters, the limits of detection (LODs), and the limits of quantification (LOQs) of procyanidins in the HPLC-FLD method.

Analyte	Conc. Range (μg/mL)	Calibration Curve Equation	Correlation Coefficient	LOD (ppb)	LOQ (ppb)
Catechin	0.5–100	y = 10^7^x + 6710	0.9957	87.80 ± 34.80	263.40 ± 104.4
Epicatechin	0.5–50	y = 9 × 10^6^x + 1458	0.9981	42.80 ± 6.47	128.40 ± 19.41
Procyanidin B1	0.5–100	y = 5 × 10^6^x − 7107	0.9958	280.00 ± 5.26	840.00 ± 15.78
Procyanidin B2	0.5–100	y = 5 × 10^6^x − 2474	0.9999	130.00 ± 23.40	390.00 ± 70.20
Procyanidin A2	0.5–100	y = 10^7^x − 4400	0.9997	130.00 ± 0.10	390.00 ± 0.30
Procyanidin C1	0.5–100	y = 5 × 10^6^x − 3264	0.9998	69.50 ± 43.10	208.50 ± 129.3

Concentration, the limit of detection (LOD), and the limit of quantification (LOQ).

**Table 4:** The HPLC-FLD method validation results.

Analyte	Conc. (μg/mL)	Precision (% CV)		Accuracy (% bias)	
		Intraday	Interday	Intraday	Interday
	1	6.5	5.7	−0.08	−0.08
Catechin	10	7.2	5.5	−0.20	−0.90
	100	3.7	3.6	−0.32	0.05
	1	4.8	4.6	−0.05	−0.05
Epicatechin	10	6.3	4.3	−0.29	−0.23
	100	4.2	2.5	−0.83	−0.60
	1	1.3	13.9	0.07	0.08
Procyanidin B1	10	3.7	6.4	−0.30	−0.27
	100	0.8	2.8	−3.59	−3.46
	1	7.4	12.8	−0.01	−0.01
Procyanidin B2	10	0.7	10.1	−0.32	−0.24
	100	3.4	4.1	−0.32	−0.37
	1	10.3	6.7	−0.02	−0.02
Procyanidin A2	10	8.7	6.1	−0.48	−0.45
	100	4.4	4.5	−4.00	−4.21
	1	8.8	6.5	−0.01	−0.01
Procyanidin C1	10	4.8	5.5	−0.48	−0.48
	100	1.0	2.5	−3.50	−3.70

**Table 5:** Extraction method validation results of the recovery (%) and matrix effect (%).

Analyte	Conc. (μg/mL)	Recovery (%)	Matrix Effect (%)
	10	92.23	8.53
Catechin	25	83.95	−17.64
	50	87.32	−13.04
	10	95.84	5.26
Epicatechin	25	106.51	−19.71
	50	79.43	2.37
	2.5	112.02	19.47
Procyanidin B1	5	85.28	−14.89
	10	85.87	−18.87
	2.5	79.92	−18.54
Procyanidin B2	5	87.87	−19.14
	10	94.72	19.79
	25	84.20	−13.38
Procyanidin A2	50	104.25	−14.66
	100	93.48	−1.25
	2.5	106.46	19.66
Procyanidin C1	5	103.78	−17.56
	10	98.02	19.93

**Table 6:** Total polyphenol and total flavonoid content of the matrices.

Herbal Matrix	Polyphenol Content (mg GAE/g DW) *	Total Flavonoid Content (mg CAT/g DW) **
*Sambucus nigra* L. flower	134.10 ± 6.36 ^c^	26.17 ± 2.54 ^b^
*Sambucus nigra* L. leaves	71.31 ± 2.12 ^d^	15.01 ± 2.23 ^c^
*Crataegus monogyna* Jacq. flowers and leaves	82.85 ± 4.37 ^d^	4.74 ± 0.16 ^d^
*Peumus boldus* Molina leaves	266.52 ± 22.35 ^b^	39.57 ± 2.80 ^a^
*Eleutherococcus senticosus* Maxim. root	6.23 ± 1.48 ^d^	3.43 ± 0.48 ^d^
*Paullinia cupana* Kunth. seed	372.67 ± 15.49 ^a^	42.59 ± 2.20 ^a^
*Hamamelis virginiana* L. leaves	136.75 ± 29.81 ^c^	7.95 ± 1.28 ^d^
Grape pomace	12.65 ± 1.42 ^d^	7.18 ± 0.81 ^d^

Results are expressed as mean ± SD, and *p* < 0.05 was considered statistically significant. Statistical significance was calculated by one-way ANOVA, followed by Tukey’s post hoc test. Different letters reveal significant differences. * GAE:gallic acid equivalent; ** CAT: catechin.

**Table 7:** DPPH radical scavenging capacity for each extract.

Herbal Matrix	In-House Result (µmol Trolox/g DW)	Literature Result (µmol Trolox/g DW)	References
*Sambucus nigra* L. flower	758.89 ± 100.20 ^a,b^	570–920	[112]
*Sambucus nigra* L. leaves	374.40 ± 47.20 ^c^	104.35 ± 0.22	[115]
*Crataegus monogyna* Jacq. flowers and leaves	62.96 ± 5.27 ^d^	29–57	[113]
*Peumus boldus* Molina leaves	935.23 ± 169.85 ^a^	524.17	[28]
*Eleutherococcus senticosus* Maxim. root	5.70 ± 1.44 ^d^	Not reported	-
*Paullinia cupana* Kunth. seed	693.63 ± 48.04 ^b^	67.91 ± 4.00	[116]
*Hamamelis virginiana* L. leaves	27.34 ± 2.11 ^d^	Not reported	-
Grape pomace	196.91 ± 0.86 ^c^	120–230	[114]

Results are expressed as mean ± SD, and *p* < 0.05 was considered statistically significant. Statistical significance was calculated by one-way ANOVA followed by Tukey’s post-hoc test. Different letters reveal significant differences.

## Data Availability

The data used to support the findings of this study are included in this article.

## Supplementary Materials

The following supporting information can be downloaded at: https://www.mdpi.com/article/10.3390/antiox13050586/s1. Table S1. Effect of the methanol/water (%) ratio on the phenolic and procyanidins distribution of grape marc extract. Table S2. Effect of solid-solvent ratio (%) on the phenolic and procyanidins distribution of grape marc extract. Table S3. Effect of the formic acid content (%) on the phenolic and procyanidins distribution of grape marc extract. Table S4. Effect of the sonication time (min) on the phenolic and procyanidins distribution of grape marc extract. Table S5. Effect of the shaking time (min) on the phenolic and procyanidins distribution of grape marc extract. Table S6. Pearson correlation analysis between antioxidant assay and TFC, TPC, antioxidant activity, and procyanidin content of all experimental matrices.
