# Supplementary material for: Optimization and Validation of Procyanidins Extraction and Phytochemical Profiling of Seven Herbal Matrices of Nutraceutical Interest
Source: Antioxidants (Basel). 2024 May 10;13(5):586. doi: 10.3390/antiox13050586 (PMC11117784; doi:10.3390/antiox13050586)
Supplement: Supplementary file 1 [file antioxidants-13-00586-s001.zip › antioxidants-2983024-supplementary.pdf]

Table S1. Effect of the methanol/water (%) ratio on the phenolic and procyanidins distribution of grape marc extract.

| MeOH/ $H_2O$ (%) | Formic acid content (%) | Dry Mass ratio (%) | Sonication time (min) | Shaking time (min) | Total polyphenolic content (mg GAE/g DW) | Catechin (µg/ g DW) | Epicatechin (µg/ g DW) | Procyanidin B1 (µg/ g DW) | Procyanidin C1 (µg/ g DW) |
|---|---|---|---|---|---|---|---|---|---|
| 100/ 0 | 1 | 100 | 10 | 10 | 6.42 ± 0.61 [a] | 76.11 ± 7.02 [b] | 19.70 ± 3.76 [a] | ND | 28.82 ± 2.40 [a] |
| 80/ 20 | 1 | 100 | 10 | 10 | 3.59 ±0.48 [b] | 76.98 ± 10.80 [b] | 20.10 ± 2.55 [a] | 16.73 ± 2.54 [a] | 25.96 ± 3.24 [a] |
| 60/ 40 | 1 | 100 | 10 | 10 | 5.47 ± 0.29 [a] | 98.11 ± 10.37 [a] | 26.24 ± 3.38 [a] | 21.19 ± 1.77 [a] | 37.98 ± 6.43 [a] |
| 40/ 60 | 1 | 100 | 10 | 10 | 2.20 ± 0.10 [c] | 56.86 ± 10.91 [c] | 13.34 ± 3.55 [b] | 16.21 ± 2.56 [a] | 18.17 ± 3.21 [b] |
| 20/ 80 | 1 | 100 | 10 | 10 | 0.80 ± 0.07 [d] | 41.20 ± 2.04 [c] | 9.68 ± 1.32 [b] | 13.32 ± 0.53 [b] | 14.32 ± 2.53 [b] |
| 0/ 100 | 1 | 100 | 10 | 10 | 0.42 ±0.03 [d] | ND | 13.77 ± 0.02 [b] | ND | ND |

Results are expressed as mean ± SD, and $p < 0.05$ was considered statistically significant. Statistical significance was calculated by one-way ANOVA followed by Tukey's post-hoc test. Different letters reveal significant differences. "ND" means "not detected".

Table S2. Effect of solid-solvent ratio (%) on the phenolic and procyanidins distribution of grape marc extract.

| MeOH/ $H_2O$ (%) | Formic acid content (%) | Sonication time (min) | Shaking time (min) | Solid solvent ratio (%) | Total polyphenolic content (mg GAE/g DW) | Catechin (µg/ g DW) | Epicatechin (µg/ g DW) | Procyanidin B1 (µg/ g DW) | Procyanidin C1 (µg/ g DW) |
|---|---|---|---|---|---|---|---|---|---|
| 60/ 40 | 1 | 10 | 10 | 25 | 9.55 ± 1.51 [a] | 303.27 ± 12.18 [a] | 221.60 ± 5.51 [a] | 111.80 ± 2.79 [a] | 64.36 ± 3.77 [a] |
| 60/ 40 | 1 | 10 | 10 | 50 | 9.18 ± 1.61 [a] | 319.10 ± 9.12 [a] | 226.04 ± 2.92 [a] | 96.16 ± 7.97 [b] | 45.63 ± 2.96 [a] |
| 60/ 40 | 1 | 10 | 10 | 75 | 4.08 ± 0.18 [b] | 150.17 ± 3.15 [b] | 102.93 ± 7.28 [b] | 58.4 ± 3.43 [b] | 50.10 ± 4.90 [a] |
| 60/ 40 | 1 | 10 | 10 | 100 | 8.10 ± 0.60 [a] | 215.00 ± 9.28 [b] | 247.56 ± 11.82 [a] | 55.70 ± 4.04 [b] | 28.60 ± 1.55 [b] |
| 60/ 40 | 1 | 10 | 10 | 125 | 5.43 ± 1.16 [b] | 112.33 ± 4.01 [b] | 195.15 ± 4.61 [a] | 36.47 ± 5.42 [c] | 21.90 ± 3.96 [b] |
| 60/ 40 | 1 | 10 | 10 | 150 | 2.94 ± 0.59 [b] | 88.68 ± 8.53 [b] | 91.85 ± 8.69 [b] | 28.77 ± 2.46 [c] | 15.47± 1.37 [b] |

Results are expressed as mean ± SD, and $p < 0.05$ was considered statistically significant. Statistical significance was calculated by one-way ANOVA followed by Tukey's post-hoc test. Different letters reveal significant differences. "ND" means "not detected".

Table S3. Effect of the formic acid content (%) on the phenolic and procyanidins distribution of grape marc extract.

| MeOH/ $H_2O$ (%) | Formic acid content (%) | Sonication time (min) | Shaking time (min) | Solid solvent ratio (%) | Total polyphenolic content (mg GAE/g DW) | Catechin (µg/ g DW) | Epicatechin (µg/ g DW) | Procyanidin B1 (µg/ g DW) | Procyanidin C1 (µg/ g DW) |
|---|---|---|---|---|---|---|---|---|---|
| 60/ 40 | 0 | 10 | 10 | 100 | 4.37 ± 1.15 [b] | 32.84 ± 3.40 [c] | 23.03 ± 1.90 [b] | 20.46 ± 1.00 [b] | 15.46 ± 1.74 [b] |
| 60/ 40 | 0.5 | 10 | 10 | 100 | 4.46 ±0.93 [b] | 73.35 ± 3.70 [b] | 45.53 ± 3.08 [b] | 24.87 ± 0.89 [b] | 13.06 ± 0.01 [b] |
| 60/ 40 | 1 | 10 | 10 | 100 | 8.10 ± 0.60 [a] | 215.00 ± 9.28 [a] | 247.56 ± 11.82 [a] | 55.70 ± 4.04 [a] | 28.60 ± 1.55 [a] |
| 60/ 40 | 1.5 | 10 | 10 | 100 | 4.36 ± 0.64 [b] | 97.127 ± 5.96 [b] | 64.97 ± 0.90 [b] | 22.78 ± 0.75 [b] | 22.46 ± 4.20 [a] |
| 60/ 40 | 2 | 10 | 10 | 100 | 5.15 ± 1.30 [b] | 89.89 ± 1.88 [b] | 60.26 ± 1.32 [b] | 30.69 ± 2.85 [b] | 23.50 ± 4.35 [a] |

Results are expressed as mean ± SD, and $p < 0.05$ was considered statistically significant. Statistical significance was calculated by one-way ANOVA followed by Tukey's post-hoc test. Different letters reveal significant differences. "ND" means "not detected".

Table S4. Effect of the sonication time (min) on the phenolic and procyanidins distribution of grape marc extract.

| MeOH/ $H_2O$ (%) | Formic acid content (%) | Sonication time (min) | Shaking time (min) | Solid solvent ratio (%) | Total polyphenolic content (mg GAE/g DW) | Catechin (µg/ g DW) | Epicatechin (µg/ g DW) | Procyanidin B1 (µg/ g DW) | Procyanidin C1 (µg/ g DW) |
|---|---|---|---|---|---|---|---|---|---|
| 60/ 40 | 1 | 0 | 10 | 100 | 2.30 ± 0.54 [b] | 78.62 ± 2.31[b] | 103.84 ± 2.21 [c] | 28.85 ± 2.84 [b] | 15.13 ± 2.40 [a] |
| 60/ 40 | 1 | 5 | 10 | 100 | 5.36 ± 0.36 [b] | 124.03 ± 5.71[b] | 150.86 ± 5.51 [b] | 38.74 ± 1.24 [b] | 20.32 ± 0.45 [a] |
| 60/ 40 | 1 | 10 | 10 | 100 | 8.10 ± 0.60 [a] | 215.00 ± 9.28 [a] | 247.56 ± 11.82 [a] | 55.70 ± 4.04 [a] | 28.60 ± 1.55 [a] |
| 60/ 40 | 1 | 15 | 10 | 100 | 7.50 ± 1.94 [a] | 228.00 ± 9.71 [a] | 241.99 ± 9.75 [a] | 57.68 ± 7.48 [a] | 27.84 ± 4.96 [a] |
| 60/ 40 | 1 | 20 | 10 | 100 | 6.27 ± 0.23 [a] | 227.72 ± 9.46 [a] | 236.45 ± 6.93 [a] | 60.18 ± 2.10 [a] | 30.71± 1.78 [a] |

Results are expressed as mean ± SD, and $p < 0.05$ was considered statistically significant. Statistical significance was calculated by one-way ANOVA followed by Tukey's post-hoc test. Different letters reveal significant differences. "ND" means "not detected".

Table S5. Effect of the shaking time (min) on the phenolic and procyanidins distribution of grape marc extract.

| MeOH/ $H_2O$ (%) | Formic acid content (%) | Sonication time (min) | Shaking time (min) | Solid solvent ratio (%) | Total polyphenolic content (mg GAE/g DW) | Catechin (μg/ g DW) | Epicatechin (μg/ g DW) | Procyanidin B1 (μg/ g DW) | Procyanidin C1 (μg/ g DW) |
|---|---|---|---|---|---|---|---|---|---|
| 60/ 40 | 1 | 10 | 0 | 100 | 4.33 ± 0.03 [b] | 86.96 ± 6.60 [b] | 48.81 ± 2.47 [b] | 29.26 ± 0.56 [b] | 23.30 ± 2.18 [a] |
| 60/ 40 | 1 | 10 | 5 | 100 | 3.57 ± 0.28 [b] | 91.43 ± 7.10 [b] | 60.35 ± 2.54 [b] | 33.72 ± 2.92 [b] | 16.80 ± 2.29 [a] |
| 60/ 40 | 1 | 10 | 10 | 100 | 8.10 ± 0.60 [a] | 215.00 ± 9.28 [a] | 247.56 ± 11.82 [a] | 55.70 ± 4.04 [a] | 28.60 ± 1.55 [a] |
| 60/ 40 | 1 | 10 | 15 | 100 | 9.65 ± 0.89 [a] | 187.71 ± 10.86 [a] | 150.29 ± 2.84 [a] | 53.96 ± 4.59 [a] | 24.91 ± 1.24 [a] |
| 60/ 40 | 1 | 10 | 20 | 100 | 3.85 ± 1.07 [b] | 128.74 ± 2.01[b] | 90.36 ± 9.00 [b] | 50.08 ± 3.73 [a] | 23.96 ± 1.40 [a] |

Results are expressed as mean ± SD, and $p < 0.05$ was considered statistically significant. Statistical significance was calculated by one-way ANOVA followed by Tukey's post-hoc test. Different letters reveal significant differences. "ND" means "not detected".

Table S6. Pearson correlation analysis between antioxidant assay and TFC, TPC, antioxidant activity, and procyanidin content of all experimental matrices.

| Factor | | FOLIN | DPPH | Total flavonoid content | Catechin | Epicatechin | Procyanidin A2 | Procyanidin B1 | Procyanidin B2 | Procyanidin C1 |
|---|---|---|---|---|---|---|---|---|---|---|
| FOLIN | Pearson coefficient | 1 | 0.72 | 0.91 | 0.94 | 0.76 | 0.75 | 0.93 | 0.51 | 0.56 |
| | *p*-value | - | 0.045 | 0.002 | 0.001 | 0.030 | 0.032 | 0.001 | 0.190 | 0.150 |
| DPPH | Pearson coefficient | 0.72 | 1 | 0.93 | 0.66 | 0.32 | 0.32 | 0.51 | 0.04 | 0.30 |
| | *p*-value | 0.045 | - | 0.001 | 0.045 | 0.440 | 0.440 | 0.020 | 0.920 | 0.470 |
| Total flavonoid | Pearson coefficient | 0.91 | 0.93 | 1 | 0.87 | 0.60 | 0.50 | 0.78 | 0.28 | 0.42 |

| | | | | | | | | | | |
|---|---|---|---|---|---|---|---|---|---|---|
| content | | | | | | | | | | |
| | *p*-value | 0.002 | 0.001 | - | 0.005 | 0.034 | 0.040 | 0.023 | 0.500 | 0.300 |
| Catechin | Pearson coefficient | 0.94 | 0.66 | 0.87 | 1 | 0.75 | 0.75 | 0.95 | 0.48 | 0.57 |
| | *p*-value | 0.001 | 0.075 | 0.075 | - | 0.031 | 0.032 | 0.001 | 0.230 | 0.140 |
| Epicatechin | Pearson coefficient | 0.76 | 0.32 | 0.60 | 0.75 | 1 | 1.00 | 0.83 | 0.83 | 0.46 |
| | *p*-value | 0.030 | 0.440 | 0.120 | 0.031 | - | 0.001 | 0.011 | 0.010 | 0.250 |
| Procyanidin A2 | Pearson coefficient | 0.75 | 0.32 | 0.60 | 0.75 | 1.00 | 1 | 0.83 | 0.80 | 0.41 |
| | *p*-value | 0.032 | 0.440 | 0.120 | 0.032 | 0.001 | - | 0.011 | 0.016 | 0.320 |
| Procyanidin B1 | Pearson coefficient | 0.93 | 0.51 | 0.78 | 0.95 | 0.83 | 0.83 | 1 | 0.53 | 0.45 |
| | *p*-value | 0.001 | 0.200 | 0.023 | 0.001 | 0.011 | 0.011 | - | 0.180 | 0.260 |
| Procyanidin B2 | Pearson coefficient | 0.51 | 0.04 | 0.28 | 0.48 | 0.83 | 0.80 | 0.53 | 1.00 | 0.71 |
| | *p*-value | 0.190 | 0.920 | 0.500 | 0.230 | 0.010 | 0.016 | 0.180 | - | 0.048 |
| Procyanidin C1 | Pearson coefficient | 0.56 | 0.30 | 0.42 | 0.57 | 0.46 | 0.41 | 0.45 | 0.71 | 1 |
| | *p*-value | 0.150 | 0.470 | 0.300 | 0.140 | 0.250 | 0.320 | 0.260 | 0.048 | - |
